# Idiopathic pulmonary fibrosis in 2011: key updates on guidelines and therapeutics

**DOI:** 10.1186/1465-9921-14-S1-S1

**Published:** 2013-04-16

**Authors:** U Costabel, C Albera, J Behr, V Cottin, A Guenther, L Richeldi

**Affiliations:** 1 University Hospital Ruhrlandklinik Essen, Germany; 2 University of Turin, San Luigi Gonzaga Medical School, Department of Clinical and Biological Sciences, Center for Rare Pulmonary Disease, Italy; 3 Dept. Internal Medicine III, Respiratory and Critical Care Medicine, University Hospital Bergmannsheil, Ruhr-University Bochum, Germany; 4 Hospices Civils de Lyon, Hôpital Louis Pradel, Service de pneumologie – Centre de référence national des maladies pulmonaires rares, Lyon, France; 5 Universities of Giessen and Marburg Lung Center (UGMLC), Germany; 6 Centre for Rare Lung Disease, University of Modena and Reggio Emilia, Modena, Italy

## Introduction

This first Advancing in Idiopathic pulmonary fibrosis Research (AIR) meeting took place in Berlin, on the 4^th^ and 5^th^ of November 2011, bringing leading experts in the field of Idiopathic Pulmonary Fibrosis (IPF) and European clinicians together to share knowledge on recent advances and practical experience in the management of this devastating disease.

It is widely recognised that although IPF is a rare fibrosing lung disease it is associated with a tremendous burden. The estimated median survival of patients with IPF is 2–5 years following diagnosis, and the mortality rate is greater than that associated with numerous malignancies [1,2].

This has presented significant challenges in patient management and until recently there were no pharmacological treatments approved for patients with IPF in Europe.

The timing of this meeting was therefore particularly relevant as there were a number of important advances and new findings during 2011, which included the publication of the updated guidelines from the ATS/ERS/JRS/ALAT Committee and the approval of pirfenidone for the treatment of mild-to-moderate IPF in Europe [3,4].

In addition, a press release from the National Institutes of Health reported that one arm of an ongoing trial, PANTHER-IPF, had been stopped because patients with IPF receiving a currently used triple-drug therapy consisting of prednisone, azathioprine, and N-acetylcysteine (NAC) had worse outcomes than those who received matching placebo. The interim results from PANTHER-IPF have since been published [5].

This supplement to *Respiratory Research* reported the proceedings from this first European IPF meeting, with key articles selected by the AIR Scientific Committee, which were considered essential reading for all clinicians managing patients with IPF in Europe.

Articles in this supplement included a review of the recently updated ATS/ERS/JRS ALAT diagnostic criteria by Professor Athol Wells, and discussion on how these can facilitate early diagnosis of IPF. It also considered issues that may remain unclear despite the updated guidance. Linked to the diagnosis of IPF, Nicola Sverzellati gave a master class on HRCT imaging and practical requirements for optimising CT scanning techniques.

Presentations then focused on the management of IPF. Professor Luca Richeldi discussed the Cochrane Collaboration approach to analysing clinical trial data and the findings of Cochrane meta-analyses that have been performed to determine the effect of treatments used in the management of IPF. This was followed by Professor Vincent Cottin, who summarised the outcomes from prior clinical trials in IPF and the recently published Phase III studies of pirfenidone that led to its approval in Europe. Following these updates, Professor Jürgen Behr and Luca Richeldi discussed how these data can be interpreted in light of the updated recommendations of the ATS/ERS/JRS/ALAT Committee regarding treatment of IPF. Professor Carlo Albera outlined the implications of the press release from the NIH regarding the PANTHER-IPF study.

Hopefully the selected proceedings from this meeting will provide IPF clinicians with new insights and support in the management of their patients. The intention is for the AIR meeting to become a fixed date in the calendar for respiratory physicians and researchers dedicated to improving knowledge and bringing new hope for IPF patients.

## Competing interests

U. Costabel has received consultancy fees from Actelion, Boehringer Ingelheim, Centocor, Gilead and InterMune, and has received lecture fees from InterMune.

J. Behr has received fees for speaking from Actelion, Bayer-Schering, Boehringer-Ingelheim, Encysive, GSK, Pfizer, Lilly, Nycomed, InterMune, Novartis, MSD and has served as consultant/advisor for Actelion, Bayer-Schering-Pharma, Lilly, Pari-Pharma, GSK, Pfizer, Optima, Gilead. J. Behr has also received research grants from Actelion, Bayer-Schering, InterMune, Pari-Pharma and attending international and national congresses sponsored by Actelion, Boehringer-Ingelheim, AstraZeneca, Bayer.

V. Cottin has received fees for speaking from InterMune, Boehringer Ingelheim, and Actelion, and has participated as a member of steering committees, a member of data safety monitoring boards or as an investigator to clinical trials sponsored by Actelion, Boehringer Ingelheim, Gilead, and InterMune Inc.

L. Richeldi has received consultancy fees from Boehringer Ingelheim, InterMune, Celgene and Gilead, and lecture fees from InterMune.

## Acknowledgements

The authors thank C. Trenam, I. Mandic and M. Smith of IntraMed Communications for editorial assistance in the preparation of the manuscript. Development of this article was supported by InterMune AG.

## Declarations

This article has been published as part of *Respiratory Research* Volume 14 Supplement 1, 2013:IPF in 2011: Key updates on guidelines and therapeutics. The full contents of the supplement are available online at http://respiratory-research.com/supplements/14/S1. Publication of this supplement was supported by IntraMed Communications with funding from InterMune, AG. InterMune is the manufacturer of pirfenidone, a product mentioned in this article. The supplement originates from presentations given at the “AIR Event: Advancing IPF Research. Working together to translate IPF research into practice” held in Berlin in November 2011. The publication was proposed by IntraMed Communications and developed in consultation with the journal. All articles in the supplement have undergone the journal’s standard peer review process.
